# Odor awareness modulates the association between perceived stress and chemosensory anhedonia in women

**DOI:** 10.1002/pchj.769

**Published:** 2024-05-16

**Authors:** Qian Hu, Muyesaier Tuluhong, Pengfei Han

**Affiliations:** ^1^ Faculty of Psychology Southwest University Chongqing China; ^2^ MOE Key Laboratory of Cognition and Personality Southwest University Chongqing China

**Keywords:** chemosensory anhedonia, females, odor awareness, perceived stress, sex difference

## Abstract

Chronic stress alters reward sensitivity and contributes to anhedonia. Chemosensation is dominated by a hedonic dimension, but little is known about the association between chronic perceived stress and hedonic chemosensation in non‐clinical populations. In the current study, 325 participants (201 females) completed a questionnaire‐based survey measuring their chronic perceived stress (Perceived Stress Scale; PSS), chemosensory pleasure (Chemosensory Pleasure Scale; CPS), and olfactory metacognitive abilities (odor awareness, affective impact of odor, importance of olfaction). For females, higher PSS scores significantly predicted lower CPS scores, which is mediated by the positive odor awareness. Moreover, negative odor awareness was identified as a moderator underlying the relationship between PSS and CPS scores in females  but not in males. For females, higher PSS predicted lower CPS for those with lower, but not for those with higher levels of negative odor awareness. These results show that the link between chronic perceived stress and chemosensory anhedonia is pronounced in females, with olfactory perception playing a key role. The current study provides insights into the understanding of stress‐related anhedonia and into the development of effective treatments.

## INTRODUCTION

Chronic stress is well known for its detrimental effects on cognitive, emotional, and physiological functions. Perceived stress refers to the degree to which an individual finds the circumstances in their life to be unpredictable, uncontrollable, and/or overwhelming (Harris et al., 2023). In otherwise healthy populations, perceived stress influences the pathogenesis of physical disease by inducing negative affective states and serving as a significant risk factor for the development of conditions such as anxiety and depression (Hammen, 2005; Melchior et al., 2007). Research across species, including rodents and humans, has shown that exposure to chronic stressors can lead to impairments in reward function, resulting in anhedonic‐like behavior (Ironside et al., 2018; Stanton et al., 2019). For example, studies in rats and mice have reported decreased sucrose preference and/or diminished social interaction following chronic stress exposure (Willner, 2005). In humans, high self‐reported perceived stress is associated with blunted hedonic capacity or sensitivity to reward cues (food or money) (Ferreira et al., 2019; Luckett et al., 2015; Pizzagalli et al., 2007).

Odor perception is dominated by a hedonic dimension, with the most salient attribute of odors being pleasantness or unpleasantness (Yeshurun & Sobel, 2010). In animal models, studies have shown long‐lasting effects of chronic stress on the olfactory (chemosensory) system (Bombail, 2019; Yuan et al., 2015), leading to significant olfactory deficits and increased anhedonia, as indicated by reduced preferences for palatable sweet tastes (Muscat & Willner, 1992; Vaz et al., 2018) or reduced responses to pleasant odors (Athanassi et al., 2023; Raynaud et al., 2015). However, there has been little empirical evidence from human participants demonstrating the relationship between chronic stress and anhedonia in the chemosensory (taste and smell) domain. The Chemosensory Pleasure Scale (CPS) is a 3‐factor scale, developed to measure the experience of anticipatory, natural, and food‐related chemosensory pleasure in non‐clinical participants (Zhao et al., 2019). Apart from the CPS, several psychometric tools have been developed to measure metacognitive abilities in the chemosensory domain (mainly olfaction; Han et al., 2021). These questionnaires assess the cognitive and affective experience of or attitude towards olfactory experiences using global, retrospective ratings, such as the Odor Awareness Scale (OAS; Smeets et al., 2008), the Affective Impact of Odor Scale (AIO; Wrzesniewski et al., 1999) and the Importance of Olfaction Questionnaire (IOQ; Croy et al., 2010). They measure a stable trait, rather than a state‐like feature, that can predict and explain how people process olfactory information and respond to situations involving olfactory cues (Smeets et al., 2008). In particular, a recent study has shown that individuals' awareness of negative and positive odors in the environment predicts their hedonic perception of real odor stimuli (Bo et al., 2023). It has been suggested that emotion (affective response) and consciousness (awareness) overlap and interact, each being necessary for aspects of the other (Tsuchiya & Adolphs, 2007). Therefore, one prediction would be that if levels of perceived stress are correlated with chemosensory hedonism, such a relationship may be modulated by olfactory metacognitive factors, as measured by the OAS, AIO, or IOQ.

The existing literature is consistent in showing sex differences in olfaction (Sorokowski et al., 2019) and in stress responses in emotion and cognition (ter Horst et al., 2012). An earlier study also proposed a model in which chronic stress induced sex‐specific neuromorphological changes in limbic structures that affect cognitive abilities and emotional arousal (McLaughlin et al., 2009). Sex‐specific functional and morphological changes associated with chronic stress exposure have been suggested in an animal study (Bittar et al., 2021). In addition, there is evidence that both stress and metacognitive factors contribute to sex differences in the perception and response to chemosensory stimuli (Ohla & Lundstrom, 2013). Women exhibit higher scores in multiple aspects of olfactory metacognitive abilities (Han et al., 2021) and general emotional awareness (Barrett et al., 2000; Wright et al., 2018). Awareness is directed towards the hedonic value of odors and olfactory mental imagery (Zhou et al., 2022), which has an emotional element (Bensafi & Rouby, 2007). Thus, it is plausible to explore whether there is a sex‐specific pattern in the association between perceived stress and olfactory hedonism.

The primary aim of the present study was to investigate the association between subjectively perceived stress and chemosensory hedonism in a non‐clinical population. We hypothesized that higher levels of perceived stress is associated with decreased chemosensory hedonism. In addition, the study was aimed to examine the  role of olfactory metacognitive abilities in the association between perceived stress and chemosensory hedonism and the potential gender differences. Given its exploratory nature, the second research objective has no specific hypothesis. 

## MATERIALS AND METHODS

### Participants

A total of 433 college students recruited from the local university campus participated in the study. Exclusion criteria were known olfactory disorders, nasal congestion, rhinitis, neurological or mental illness, smoking, and medication that may affect olfactory functions. The experiment was conducted according to the Helsinki Declaration on biomedical research with human participants. The experimental protocol had the approval of the Ethics Committee of the Department of Psychology, Southwest University (H23150). All participants signed an informed consent form before completing the questionnaires.

### Procedure

Participants completed an online survey that included personal information, the Perceived Stress Scale (PSS), the Chalder Fatigue Scale (CFS), the Beck Depression Inventory (BDI), the Trait Anxiety Subscale of the State‐Trait Anxiety Inventory (STAI‐T), the Big Five Inventory Neuroticism subscale (BFI‐N), the Chemosensory Pleasure Scale (CPS), the Affective Impact of Odor (AIO), the Odor Awareness Scale (OAS), and the Importance of Olfaction (IOQ). To counterbalance the order of the questionnaires, half of the participants (*n* = 224) completed the stress‐related emotion questionnaires first (PSS, CFS, BDI, STAI‐T, BFI‐N) andthe olfaction‐related questionnaires (CPS, AIO, OAS, IOQ). The other half of the participants (*n* = 209) completed the questionnaires in reverse order. There are also three additional polygraph questions in the total questionnaire.

### Questionnaires

#### Perceived Stress Scale

To assess the subjective experience of chronic stress, participants completed the Chinese version of the PSS (Lu et al., 2017). The 10‐item scale is used to assess how out of control, unpredictable, or overloaded an individual's life has felt in the past month on a 5‐point scale (0 = *never*, 4 = *always*) (Cohen et al., 1983). The total score on the scale is the sum of the scores on each item (0–40). The higher the PSS score, the higher the level of daily stress. The Cronbach *α* coefficient of the questionnaire was .794.

#### Chalder Fatigue Scale

The CFS was used to measure participants' fatigue, weakness, or lack of energy in the past month (Cella & Chalder, 2010). The 11‐item CFS measures fatigue‐related symptoms in two dimensions – physical and psychological fatigue – and is scored on a 4‐point scale (0 = *less fatigued than usual*, 1 = *no more fatigued than usual*, 2 = *more fatigued than usual*, 3 = *much more fatigued than usual*). Higher scores on each dimension indicate higher levels of fatigue. The Cronbach *α* coefficient of the questionnaire was .921.

#### Beck Depression Inventory

The Chinese version of the BDI was administered to measure the depressive tendency of the participants (Shek, 1990). The scale is made up of 13 questions, each with four short phrases representing four possible responses. The Cronbach *α* coefficient of the questionnaire was .948.

#### Trait Anxiety subscale of the State‐Trait Anxiety Inventory

The Chinese version of the STAI‐T was adapted (Shek, 1993) to assess differences between people in their tendency to perceive and react to stressful situations as threatening (Spielberger, 1983). The Inventory consists of 20 items, and each item is scored on a 4‐point scale (1 = *almost never*, 2 = *sometimes*, 3 = *often*, 4 = *almost all the time*). A higher score indicates a higher level of trait anxiety. The Cronbach *α* coefficient of the questionnaire was .890.

#### Neuroticism subscale of the Big Five Inventory

The Neuroticism subscale of the Big Five Inventory was used to assess participants' emotional regulation and emotional instability (McCrae & Costa, 1987). The revised Chinese version of this scale has been extensively tested for reliability and validity in the context of Chinese culture (Chan et al., 2011). The subscale consists of 12 items rated on a 5‐point scale from 1 (*strongly disagree*) to 5 (*strongly agree*). The Cronbach *α* coefficient of the questionnaire was .866.

#### Chemosensory hedonism

The CPS measures hedonic olfactory and gustatory capacities and is a reliable and valid method for assessing an individual's ability to smell and taste pleasure in non‐clinical samples (Zhao et al., 2019). It consists of 12 items, including three factors – food (ability to experience pleasure when eating), imagination (ability to experience pleasure when anticipating food), and nature (ability to experience pleasure when smelling natural scents). The Cronbach *α* coefficient of the questionnaire was .915.

#### Affective impact of odor

The AIO measures the role of odors in daily life, especially the impact of good and bad odors in influencing favorability and memory of places, things, and people (Wrzesniewski et al., 1999). The Cronbach *α* coefficient of the questionnaire was .789.

#### Odor Awareness Scale

The OAS assesses people's tendency to pay attention to odors in the environment, including food, civilisation, nature, and human sources (Smeets et al., 2008). The OAS consists of 32 items, which are divided into two factors: (1) a positive factor (11 items, OAS_pos) representing pleasant or attractive odors that can be safely approached, and (2) a negative factor (21 items, OAS_neg) representing dangerous or unpleasant odors that are best avoided. The Cronbach α coefficient of the questionnaire was .889.

#### Importance of Olfaction

The IOQ consists of 20 items for which participants are presented with statements about olfaction (Croy et al., 2010), which are answered on a 4‐point Likert scale (0 = *strongly disagree*, 3 = *strongly agree*). There are three subscales (application, association, and outcome). The application subscale (6 items) reflects the extent to which people use olfaction in daily activities; the association subscale (6 items) considers emotions, memories, and events triggered by olfaction; the outcome subscale (6 items) reflects the extent to which people rely on olfaction in everyday decision‐making. The remaining two items (7, 18) tend to exaggerate the importance of olfaction. The Cronbach *α* coefficient of the questionnaire was .874.

### Data clean and statistical analysis

Data from 108 participants were excluded, with 40 participants took less than 5 min to complete the questionnaire, and 68 answered one of the three polygraph questions incorrectly. After screening, 325 participants were finally included in the analyses. First, we tested whether the variables were approximately normally distributed using Shapiro–Wilk tests. Bivariate Pearson correlations were first performed between the questionnaires, and a Bonferroni adjustment was used. The alpha level was set at *p* < .0045 (0.05/11) to avoid false positives. Pearson correlations between variables were calculated. Age and BMI were included as covariates in all analyses. All analyses were performed using SPSS (version 26.0, SPSS Inc., Chicago, IL, USA) and GraphPad Prism 8 (GraphPad Software, Inc. La Jolla, CA, USA) software.

To test the effect of perceived of stress on chemosensory metacognition and sex differences, grouping regression models (males and females, respectively) were performed using SPSS 26 software. To explore the effect of positive odor awareness on the relationship between PSS and olfactory metacognitions, mediation analyses were conducted using the PROCESS macro for SPSS (Hayes, 2013). To explore the effect of negative odor awareness in the relationship between PSS and olfactory metacognitions, moderation analyses were conducted using the PROCESS macro for SPSS with Model 1. PSS scores (the independent variable) and positive/negative odor awareness (the mediator/moderator variable) were both mean‐centered prior to the analyses. To further clarify the specific pattern of the moderation effect, a simple slope analysis of odor awareness in the relationship between PSS and CPS was conducted using the PROCESS macro. To facilitate the interpretation of significant interaction terms, perceived stress was divided into two groups, namely low PSS level and high PSS level (±1 SD around the mean). The graphical displays were produced using GraphPad Prism 8 software.

## RESULTS

### Participant characteristics

The characteristics of the study participants are shown in Table 1. There were no sex‐based differences in perceived stress, chronic fatigue, depression, or neuroticism scores. However, the male participants were older, had a higher BMI, and had lower scores for trait anxiety than did the female participants.

**TABLE 1:** Summary of sample demographic characteristics.

Variables	All, *N* = 325	Male, *n* = 124	Female, *n* = 201	Comparison *p‐*value
Age	20.8 (2.0)	21.1 (2.3)	20.6 (1.8)	**.04**
BMI	20.4 (3.6)	21.5 (4.2)	19.7 (3.0)	**<.001**
PSS	16.8 (6.4)	16.8 (6.2)	16.8 (6.4)	.92
CFS	14.1 (7.4)	13.4 (7.4)	14.6 (7.3)	.19
BDI	7.1 (7.7)	6.7 (8.2)	7.4 (7.4)	.41
STAI‐T	41.3 (9.9)	39.7 (9.6)	42.2 (10.0)	**.03**
BFI‐N	33.2 (9.1)	32.0 (9.3)	33.9 (8.9)	.07

*Note*: Data presented as mean (standard deviations). Bold values indicate statistically significant results.

Abbreviations: BDI = Beck Depression Inventory; BFI‐N = Neuroticism subscale of the Big Five Inventory; BMI = body mass index; CFS = Chalder Fatigue Scale; PSS = Perceived Stress Scale; STAI‐T = Trait Anxiety subscale of the State–Trait Anxiety Inventory.

### Correlation analysis of perceived stress and olfactory metacognition

The correlations between the variables are shown in Table 2. After adjustment for age, sex, and BMI, the PSS score was negatively correlated with the CPS total score (*r* = −.20, *p* < .001) and with the scores of two subscales of the CPS, namely the ability to enjoy food flavor (*r* = −.19, *p* = .001) and the ability to enjoy smells in nature (*r* = −0.21, *p* < .001), but was not significantly correlated with the imagination of chemosensory cues (*r* = −.13, *p* = .02). In addition, the PSS score was not correlated with the total score of the OAS or the IOQ. However, there was a significant correlation between the PSS and the olfactory application subscale of the IOQ (*r* = −.20, *p* < .001). When correlation analysis was performed separately for male and female participants, there was a significant correlation between PSS and the CPS total scores for female (*r* = −.20, *p* = .001) but not for male (*r* = −.16, *p* = .08) participants. Similarly, the correlation between PSS and positive odor awareness (OAS_pos) was significant for female participants (*r* = −.21, *p* = .003), but not for male participants (*r* = −.03, *p* = .74).

**TABLE 2:** Descriptive statistics of and correlations between study variables.

Variables	Age	BMI	PSS	CFS	BDI	STAI‐T	BFI‐N	CPS	AIO	OAS	IOQ
1. Age	‐										
2. BMI	.16**	‐									
3. PSS	−.03	−.02	‐								
4. CFS	.03	−.03	.67***	‐							
5. BDI	.02	−.01	.57**	.64**	‐						
6. STAI‐T	−.04	−.03	.74***	.68**	.64***	‐					
7. BFI‐N	−.04	.03	.77***	.74**	.65***	.79***	‐				
8. CPS	−.12*	.07	−.21***	−.18**	−.31***	−.32***	−.19**	‐			
9. AIO	−.07	−.04	−.06	.01	−.06	−.16**	−.04	.46***	‐		
10. OAS	−.03	−.07	−.03	.03	.003	−.11*	.006	.47***	.66***	‐	
11. IOQ	.002	−.09	−.06	−.02	−.08	−.19**	−.06	.58***	.68***	.77***	‐
Mean	20.8	20.9	16.8	14.1	7.1	41.3	33.2	61.0	16.8	120.6	44.0
SD	2.0	4.0	6.3	7.4	7.7	9.9	9.1	7.9	3.7	15.8	7.6
Range	18–35	14.9–39.8	0–35	0–33	0–32	20–67	12–54	23–72	1–24	46–159	10–60

Abbreviations: AIO = Affective Impact of Olfaction; BDI = Becker Depression Inventory; BFI‐N = Big Five Inventory Neuroticism subscale; BMI = body mass index; CFS = Chronic Fatigue Scale; CPS = Chemosensory Pleasure Scale; IOQ = Importance of Olfaction; OAS = Odor Awareness Scale; PSS = Perceived Stress Scale; STAI‐T = Trait Anxiety subscale of the State–Trait Anxiety Inventory; asterisks indicate significant correlation without Bonferroni correction.

* *p* < .05;

** *p* < .01;

*** *p* < .001.

### Linear regression model of perceived stress to predict olfactory anhedonia

The CPS total (Female: *R* ^2^ = .045, *p* = .001; Male: *R* ^2^ = .018, *p* = .076), CPS food (Female: *R* ^2^ = .040, *p* = .002; Male: *R* ^2^ = .019, *p* = .068), and CPS imagination (Female: *R* ^2^ = .022, *p* = .02; Male: *R* ^2^ = .004, *p* = .469) were negatively predicted by PSS for female but not for male participants. The CPS nature was predicted by PSS for both female (*R* ^2^ = .038, *p =* .002) and male (*R* ^2^ = .036, *p* = .02) participants (Table 3).

**TABLE 3:** Regression analysis of perceived chronic stress on olfactory metacognition.

Model	Dependent variable	Male			Female		
		*R* ^2^	*B*	*p*	*R* ^2^	*B*	*p*
1.1	CPS	.018	−.160	.076	.045	−.224	**.001**
1.2	CPS food	.019	−.164	.068	.040	−.212	**.002**
1.3	CPS nature	.036	−.209	**.020**	.038	−.206	**.003**
1.4	CPS imagination	.004	−.066	.469	.022	−.163	**.020**

*Note*: Perceived chronic stress was used as an independent variable in all models. Bold values indicate statistically significant results.

Abbreviations: CPS = individual total chemosensory pleasure ability; CPS food = the ability to enjoy food; CPS nature = the ability to enjoy nature pleasure; CPS imagination = the ability to enjoy pleasure when anticipating food.

### Mediation effect of positive odor awareness on the relationship between perceived stress and chemosensory pleasure

**FIGURE 1:**
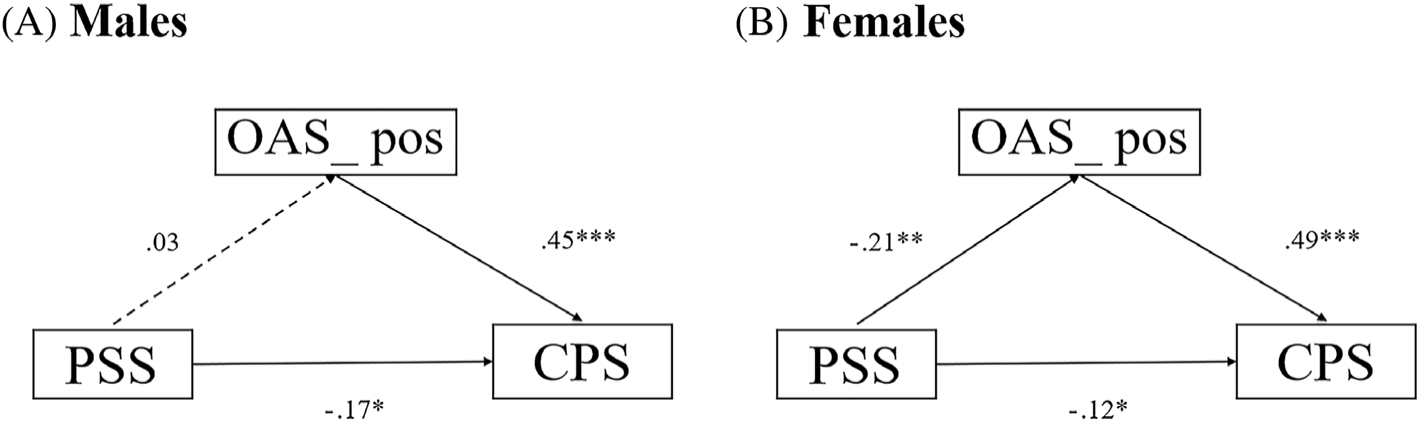
Mediation model. The effect of perceived stress on chemosensory pleasure was partially mediated by positive odor awareness in (A) males and (B) females.

For the mediation model (Table 4 and Figure 1), the PSS and CPS total were the dependent and independent variables, respectively, and the OAS and its two subscales were the mediators. One of the prerequisites for mediation was that the mediator had to be associated with the PSS. Only the OAS_pos met the criterion (see Table 4). Neither the AIO nor the IOQ showed a significant mediating effect on the association between PSS and CPS. The model showed that OAS_pos partially mediated the effect of perceived stress on chemosensory pleasure in females (*B* = −.10, 95% confidence interval CI = [−.19, −.03]) but not in males (*B* = .01, 95% CI = [−.06, .10]) (Table 4 and Figure 1). In other words, the level of perceived stress had an influence on the CPS via the pathway of its effect on OAS_pos in female participants.

**TABLE 4:** Mediating effect of positive odor awareness on the relationship between perceived stress and chemosensory pleasure.

Sex	Dependent variable	Independent variable	*B*	*t*	*p*	LLCI	ULCI	*R* ^2^	*F*
Male	OAS (M)	PSS	.10	1.08	.28	−.08	.28	.01	1.17
	CPS (Y)	PSS	−.20	−2.51	**.01**	−.36	−.04	.22	17.05***
		OAS	.44	5.49	**<.001**	.28	.60		
	OAS_neg (M)	PSS	.13	1.43	.16	−.05	.31	.02	2.03
	CPS (Y)	PSS	−.21	−2.50	**.01**	−.37	−.04	.17	12.37***
		OAS_ neg	.38	4.58	**<.001**	.22	.55		
	OAS_pos (M)	PSS	.03	0.33	.74	−.15	.21	<.01	0.11
	CPS (Y)	PSS	−.17	−2.17	**.03**	−.33	−.02	.23	17.82***
		OAS_pos	.45	5.62	**<.001**	.29	.61		
Female	OAS (M)	PSS	−.11	−1.58	.12	−.25	.03	.01	2.50
	CPS (Y)	PSS	−.17	−2.81	**.01**	−.29	−.05	.27	36.01***
		OAS	.47	7.65	**<.001**	.35	.59		
	OAS_neg (M)	PSS	−.04	−0.59	.56	−.18	.10	.01	0.34
	CPS (Y)	PSS	−.21	−3.28	**.001**	−.33	−.08	.21	25.72***
		OAS_neg	.40	6.24	**<.001**	.27	.52		
	OAS_pos (M)	PSS	−.21	−2.96	**.004**	−.34	−.07	.04	8.75**
	CPS (Y)	PSS	−.12	−2.01	**.04**	−.25	−.01	.28	38.37***
		OAS_pos	.49	7.93	**<.001**	.37	.61		

*Note*: Bold values indicate statistically significant results.

Abbreviations: M = moderators (OAS = Odor Awareness Scale; OAS_pos = Positive Odor Awareness; OAS_neg = Negative Odor Awareness); X = independent variable (PSS = Perceived Stress Scale); Y = dependent variable (CPS = Chemosensory Pleasure Scale); LLCI = lower level of confidence interval; ULCI = upper limit of confidence interval.

** *p* < .01;

*** *p* < .001.

### Moderation effect of negative odor awareness on the relationship of perceived stress and chemosensory pleasure

**FIGURE 2:**
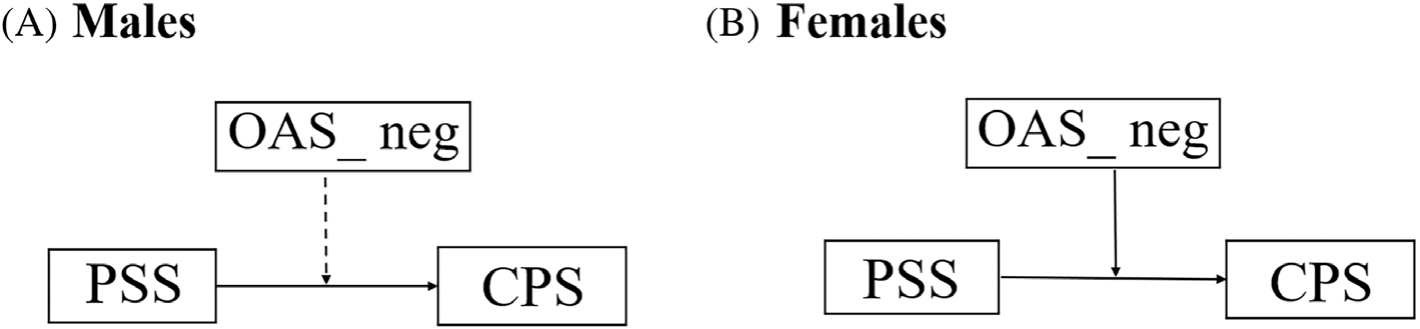
Moderation model. The moderating effect of negative odor awareness between perceived stress and chemosensory pleasure for (A) males and (B) females. Dashed line indicates non‐significant moderation effect.

In the moderation model, the total scores of the PSS and CPS were the dependent and independent variables, respectively, and the OAS and its two subscales were the moderators. The direct effect of PSS on CPS was significant for both males (*B* = −.21, *p* = .01) and females (*B* = −.23, *p* < .001) (Table 5). However, the interaction between PSS and negative odor awareness (OAS_neg) was significant only for females (*B* = .14, *p* = .02) and not for males (*B* = .03, *p* = .73) (Figure 2).

**TABLE 5:** Moderating coefficients for the effects of perceived stress and negative odor awareness acting on chemosensory pleasure.

Sex	Dependent variable	Independent variable	*B*	*t*	*p*	LLCI	ULCI
Male	CPS (M)	PSS (X)	−.21	−2.57	**.01**	−.37	−.05
		OAS (W)	.46	5.61	**<.001**	.30	.62
		Effect of X*W on M	.09	1.14	.25	−.07	.26
		F (3, 120) = 11.83, *R* ^2^ = .23, *p* < .001					
	CPS (M)	PSS (X)	−.21	−2.50	**.01**	−.38	−.04
		OAS_neg (W)	.39	4.56	**<.001**	.22	.56
		Effect of X*W on M	.03	10.34	.73	−.13	.18
		F (3, 120) = 11.83, *R* ^2^ = .23, *p* < .001					
	CPS (M)	PSS (X)	−.18	−2.25	**.03**	−.34	−.02
		OAS_pos (W)	.46	5.78	**<.001**	.30	.62
		Effect of X*W on M	.15	1.72	.09	−.02	.33
		F (3, 120) = 13.05, *R* ^2^ = .25, *p* < .001					
Female	CPS (M)	PSS (X)	−.20	−3.20	**.002**	−.32	−.08
		OAS (W)	.47	7.71	**<.001**	.35	.59
		Effect of X*W on M	.12	2.07	**.04**	.01	.24
		F (3, 197) = 25.84, *R* ^2^ = .28, *p* < .001					
	CPS (M)	PSS (X)	−.23	−3.60	**<.001**	−.35	−.10
		OAS_neg (W)	.40	6.41	**<.001**	.28	.53
		Effect of X*W on M	.14	2.29	**.02**	.02	.26
		F (3, 197) = 19.26, *R* ^2^ = .23, *p* < .001					
	CPS (M)	PSS (X)	−.15	−2.37	**.02**	−.28	−.03
		OAS_pos (W)	.49	7.98	**<.001**	.37	.61
		Effect of X*W on M	.10	1.57	.12	−.02	.22
		F (3, 197) = 26.59, *R* ^2^ = .29, *p* < .001					

*Note*: Bold values indicate statistically significant results.

Abbreviations: W, moderators (OAS = Odor Awareness Scale; OAS_pos = Positive Odor Awareness; OAS_neg = Negative Odor Awareness); X = independent variable (PSS = Perceived Stress Scale); X*W = interaction of independent variable and moderators; Y = dependent variable (CPS = Chemosensory Pleasure Scale); LLCL = lower level of confidence interval; ULLI = upper limit of confidence interval).

**FIGURE 3:**
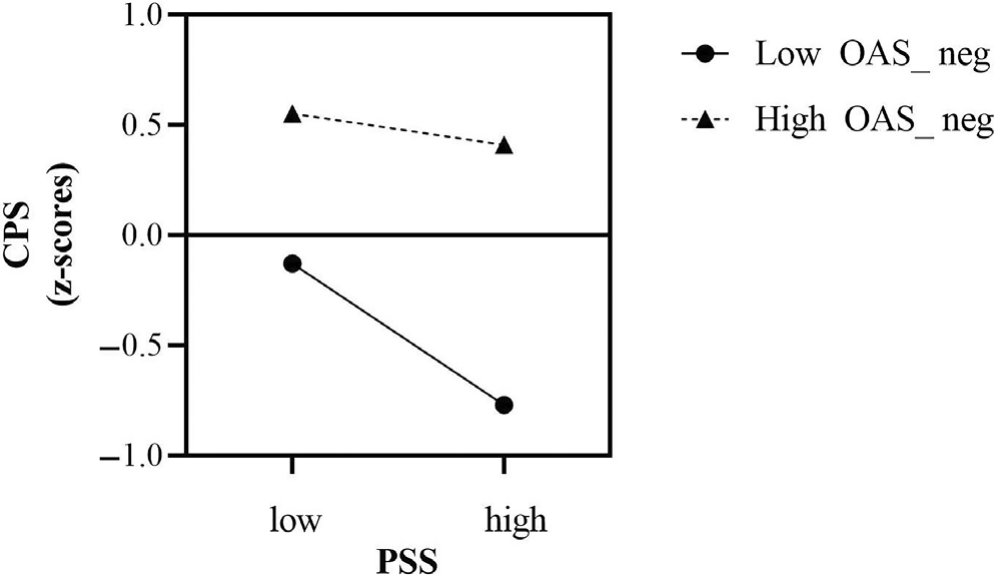
Interaction effects of perceived stress (PSS) and negative odor awareness (OAS_neg) on chemosensory pleasure (CPS) in females. Lines were plotted at −1 SD values (low) and +1 SD values (high) of negative odor awareness.

Simple slope analyses revealed that the level of perceived stress negatively predicted the CPS scores for female participants with a low OAS_neg level (*B* = − .32, *p* < .001), but not for those with a high OAS_neg level (*B* = − .09, *p* = .28) (Figure 3). In other words, a high OAS_neg buffered the negative effects of high perceived stress on chemosensory pleasure. Neither the AIO nor the IOQ showed a significant moderating effect on the relationship between PSS and CPS.

## DISCUSSION

In line with our hypothesis, higher stress perceptions were associated with reduced chemosensory hedonism in a non‐clinical population. The association between high levels of perceived stress and a reduced hedonic capacity in the non‐chemosensory domain has been reported previously (Boyle et al., 2023; Pizzagalli et al., 2007). Importantly, Pizzagalli et al. (2007) found that PSS scores predicted reduced reward responsiveness even after controlling for the overall symptoms of distress and fear, suggesting a unique effect of perceived stress on hedonic processing. Participants with higher levels of perceived stress bid lower for rewards and chose higher food bids less frequently (Ferreira et al., 2019). Another study found that participants with higher stress rated the taste of a lower‐calorie chip less hedonically and were less satisfied with their meal (Luckett et al., 2015). The CPS scores were positively correlated with hedonic ratings of odor stimuli (Bontempi et al., 2023). Thus, the reduced chemosensory hedonics in individuals with high levels of perceived stress may indicate a perceptual or behavioral change related to odor or taste perception.

The results also found sex differences in the relationship between perceived stress and olfactory‐related metacognitions. Specifically, the effect of perceived stress on CPS was significant in female, but not in male participants. Following chronic stress exposure, female mice showed more drastic changes in dopaminergic pathway structures and more anxiety‐like behaviors compared with male mice (Bittar et al., 2021). The effects of chronic stress on reduced palatable food‐seeking were found in female but not in male mice (Ball et al., 2020). In humans, there is evidence for sex‐specific effects of stress and cortisol on reward processing (Kinner et al., 2016) and brain network connectivity (Kogler et al., 2016). Taken together, the results of the present study suggest that higher perceived stress is associated with reduced chemosensory hedonism, which was more pronounced in female participants. However, the current study could not make any directional causal claims. A more detailed understanding of the causal relationship between stress and chemosensory anhedonia would require a longitudinal study.

The results showed that the level of odor awareness played a role in the relationship between perceived stress and chemosensory hedonism in female participants. Compared to men, women have more extensive knowledge of emotional experience, and higher levels of emotional awareness (Barrett et al., 2000; Wright et al., 2018). The superior emotional awareness in females may be also ture for olfactory (chemosensory) domain, because females are more often involved in olfactory activities and are exposed to a greater variety of odors (Nováková et al., 2014). First, the positive odor awareness significantly mediated the effect of PSS on CPS, so higher perceived stress may induce chemosensory anhedonia by reducing the awareness of positive odors in the environment. Individuals who pay less attention to positive odors in their daily lives may decrease the turnover rate of receptors, alter amygdala function, and ultimately reduce the ability to process olfactory cues from pleasant olfactory stimuli, resulting in anhedonia (Bo et al., 2023). In addition, perceived stress negatively affected chemosensory hedonism only in female participants who have lower levels of negative odor awareness. A recent study found that higher depressive symptoms predicted higher odor pleasantness ratings in individuals with low odor awareness, but not in those with high odor awareness (Bo et al., 2023). However, the study did not consider the positive or negative odor awareness separately. It is possible that for people who do not normally pay attention to negative odors in their daily environment, the high level of perceived stress increases the reduction in chemosensory pleasure. In contrast, those people who already pay a lot of attention to negative odors in the environment may have a characteristic negative bias of chemosensory processing, so that the different levels of perceived stress would have limited impact on the chemosensory hedonisms as assessed by the CPS. However, this speculation warrants future investigation.

Our findings on the role of odor awareness in the relationship between perceived stress and chemosensory anhedonia may provide some insights for the development of future treatments. First, odor awareness is related to the level of engagement in olfactory‐related activities (Nováková et al., 2014). Thus, increasing positive olfactory experiences would be beneficial for increasing chemosensory pleasure, as these experiences and cheosensory pleasure are strongly correlated. Second, olfactory training may be beneficial for better odor awareness and improved emotional processing (Oleszkiewicz et al., 2022), and thus may be useful for clinical populations.

Some methodological limitations of the study should be noted. First, as a widely used psychometric tool for measuring chronic stress, the PSS measures a relatively stable trait that varies slightly over short intervals and only moderately over longer intervals (Harris et al., 2023). Furthermore, it has been suggested that questionnaires measuring chronic stress are susceptible to recall bias (Weckesser et al., 2019), and self‐reported chronic stress does not satisfactorily reflect the psychophysiological (i.e., endocrine) characteristics of chronic stress to a satisfactory extent (Schmidt et al., 2020). Thus, a multi‐method and multi‐dimensional assessment of stress is needed (Dorsey et al., 2022), including the types of stressors, their controllability and severity, and the duration of stress exposure (Crosswell & Lockwood, 2020). Second, chemosensory metacognition and hedonism have only been analyzed using self‐report questionnaires. Future studies need to implement laboratory tasks specifically investigating chemosensory cognitive and emotional function. Finally, the current study focused on groups of college students with a limited sample size. Future research should expand the sample to further investigate the effect of chronic stress on chemosensory hedonism.

## CONCLUSION

In conclusion, the results of the current study showed an association between higher perceived stress and lower chemosensory (olfactory) affective hedonism, which was more pronounced in female participants. This is consistent with accumulating evidence from animal studies highlighting the link between chronic stress exposure and anhedonia. These findings also provide insight into the important role of odor awareness in linking chronic stress to chemosensory anhedonia.

## CONFLICT OF INTEREST STATEMENT

There is no potential competing interest from any of the co‐authors of the paper.

## ETHICS STATEMENT

The study was approved by the Ethics Commitee of the Department of Psychology, Southwest University (H23150). All participants provided written consent before taking the experiment.

## Data Availability

Data are available from the corresponding author under reasonable request. The self‐reported data used to support the findings of this study are restricted by the Human Research Ethics Committee at the Faculty of Psychology, Southwest University to protect participants' privacy.
